# Supplementary material for: Reconstruction of cell spatial organization from single-cell RNA sequencing data based on ligand-receptor mediated self-assembly
Source: Cell Res. 2020 Jun 15;30(9):763–78. doi: 10.1038/s41422-020-0353-2 (PMC7608415; doi:10.1038/s41422-020-0353-2)
Supplement: Supplementary file 1 — Supplementary information, Fig. S1 [file 41422_2020_353_MOESM1_ESM.pdf]

Supplementary information, Figure S1

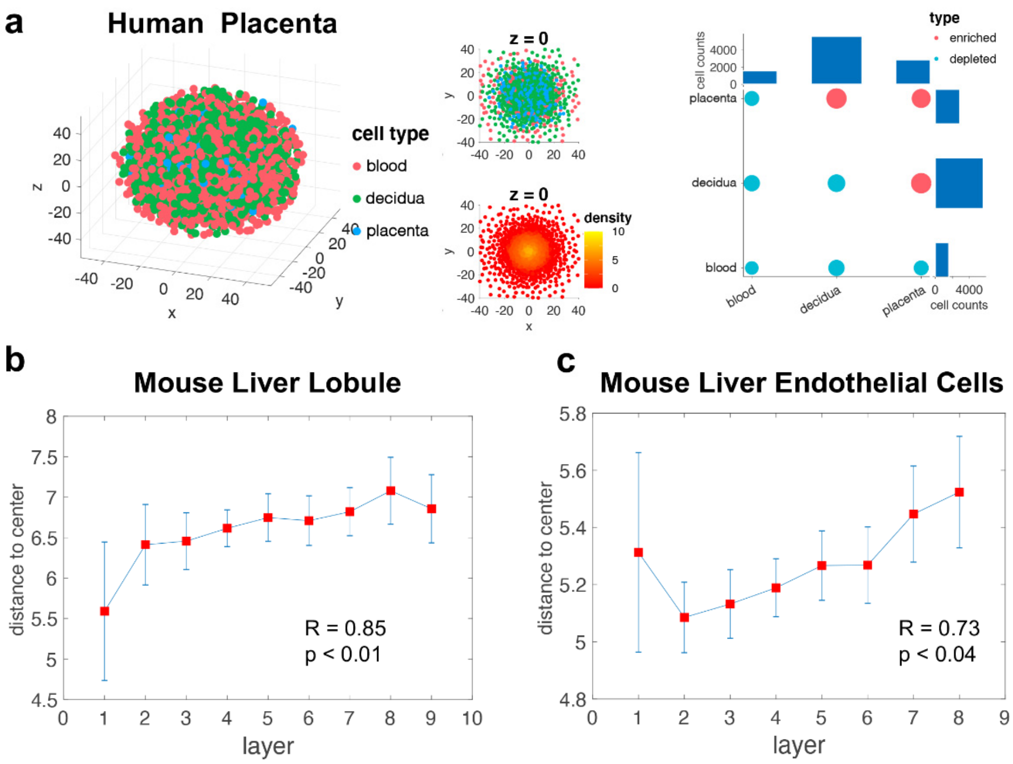

Fig. S1 Performance of CSOmap on scRNA-seq data of human placenta and mouse liver lobules. a The 3D visualization of CSOmap prediction based on the 10× genomics data of the human placenta scRNA-seq data (left), the cross-section view, and the statistical significance (right). Significant interactions between fetal placenta cells and maternal decidua cells rather than blood cells can be observed, resembling the maternal-fetal interface reported in the original paper[18]. **b** The consistence of CSOmap prediction with the annotated cell layer information (Pearson correlation) of a scRNA-seq data for mouse liver lobules[8]. **c** The consistence of CSOmap prediction based on the paired-cell sequencing data with the annotated layer information of mouse liver endothelial cells (Pearson correlation)[19]. Distance to center: Euclidean distance. Error bars: standard error. High error bars were caused by the uncertainty of the reference labels (> 93% cells having uncertainty more than 2 layers estimated by the given probabilistic distributions).
