# Supplementary material for: Reconstruction of cell spatial organization from single-cell RNA sequencing data based on ligand-receptor mediated self-assembly
Source: Cell Res. 2020 Jun 15;30(9):763–78. doi: 10.1038/s41422-020-0353-2 (PMC7608415; doi:10.1038/s41422-020-0353-2)
Supplement: Supplementary file 2 — Supplementary information, Fig. S2 [file 41422_2020_353_MOESM2_ESM.pdf]

Supplementary information, Figure S2

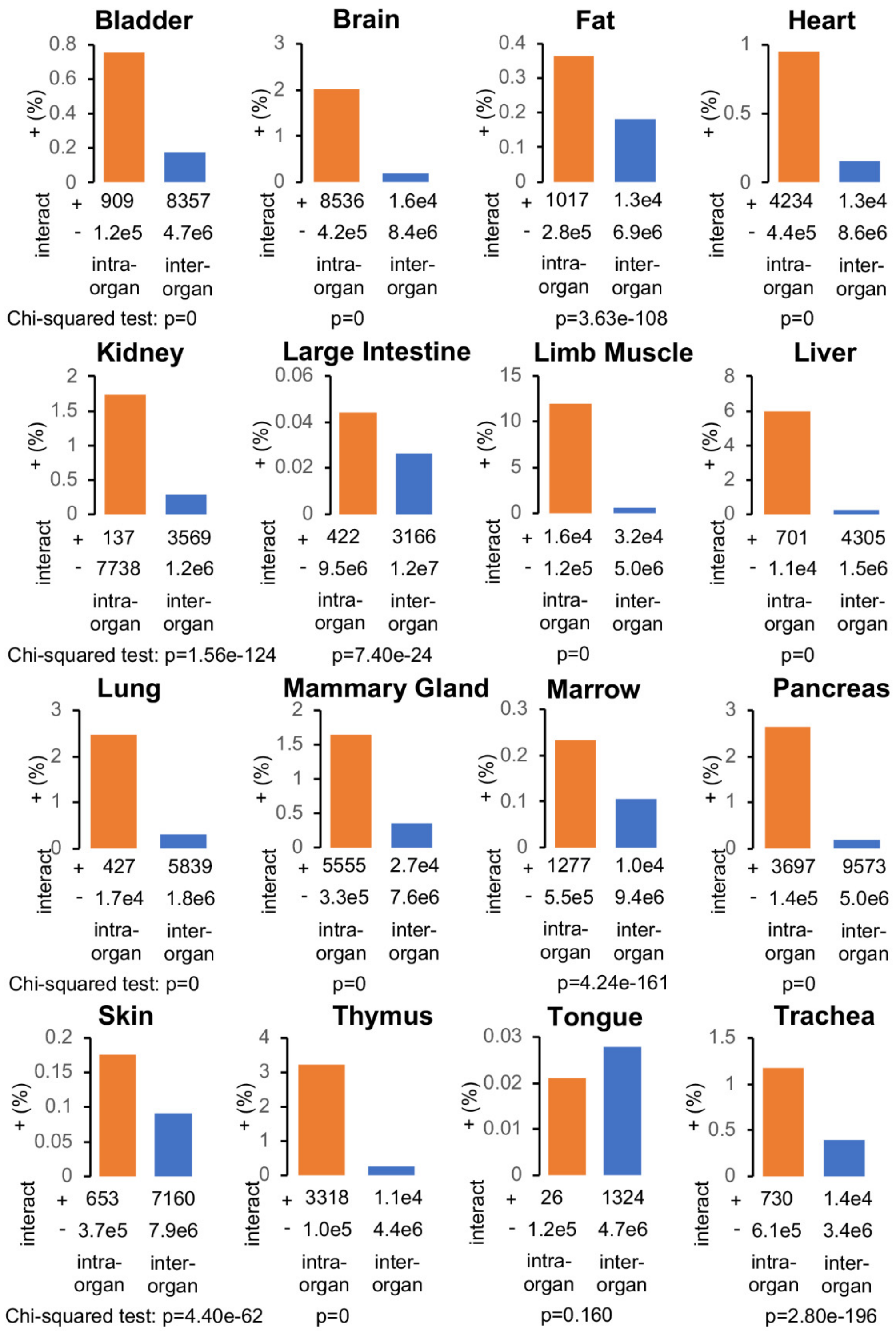

Fig. S2 CSOmap recapitulates high intra-organ cellular interactions based on the Tabula Muris dataset. For each organ, cell pairs were categorized into intra-organ

interacting pairs (Interact+Intra-organ+), intra-organ non-interacting pairs (Interact-Intra-organ+), inter-organ interacting pairs (Interact+Inter-organ+), and inter-organ non-interacting pairs (Interact-Inter-organ+), then χ^2 test was applied to examine the statistical significance of whether intra-organ cell pairs tend to have higher interacting odds than inter-organ cell pairs. The 10× genomics data were used for CSOmap prediction. Immune and neuron cells dispersing in various organs were excluded. 16 organs were retained and 15 were predicted to have higher intra-organ cellular interactions than inter-organ interactions.
