# Supplementary material for: Reconstruction of cell spatial organization from single-cell RNA sequencing data based on ligand-receptor mediated self-assembly
Source: Cell Res. 2020 Jun 15;30(9):763–78. doi: 10.1038/s41422-020-0353-2 (PMC7608415; doi:10.1038/s41422-020-0353-2)
Supplement: Supplementary file 3 — Supplementary information, Fig. S3 [file 41422_2020_353_MOESM3_ESM.pdf]

Supplementary information, Figure S3

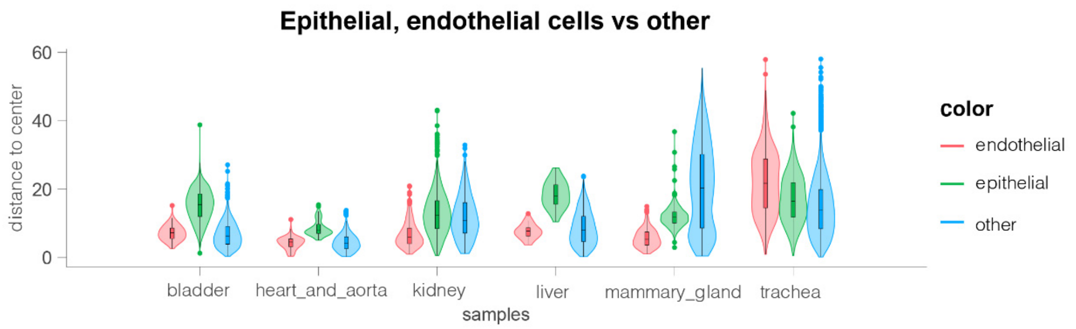

Fig. S3 CSOmap recapitulates the relative spatial locations of epithelial and endothelial cells in mouse organs based on the Tabula Muris dataset. Of six organs that have both epithelial and endothelial cells available in the Tabula Muris dataset, epithelial cells are predicted to locate in the outside space (far from the center of the pseudo-space, topologically equivalent to the organ edges) while the endothelial cells locate in the inner space (close to the center of the pseudo-space, topologically equivalent to the organ basement). Except trachea, all other organs show statistical significance based on rank-sum test.
