# Supplementary material for: Reconstruction of cell spatial organization from single-cell RNA sequencing data based on ligand-receptor mediated self-assembly
Source: Cell Res. 2020 Jun 15;30(9):763–78. doi: 10.1038/s41422-020-0353-2 (PMC7608415; doi:10.1038/s41422-020-0353-2)
Supplement: Supplementary file 4 — Supplementary information, Fig. S4 [file 41422_2020_353_MOESM4_ESM.pdf]

Supplementary information, Figure S4

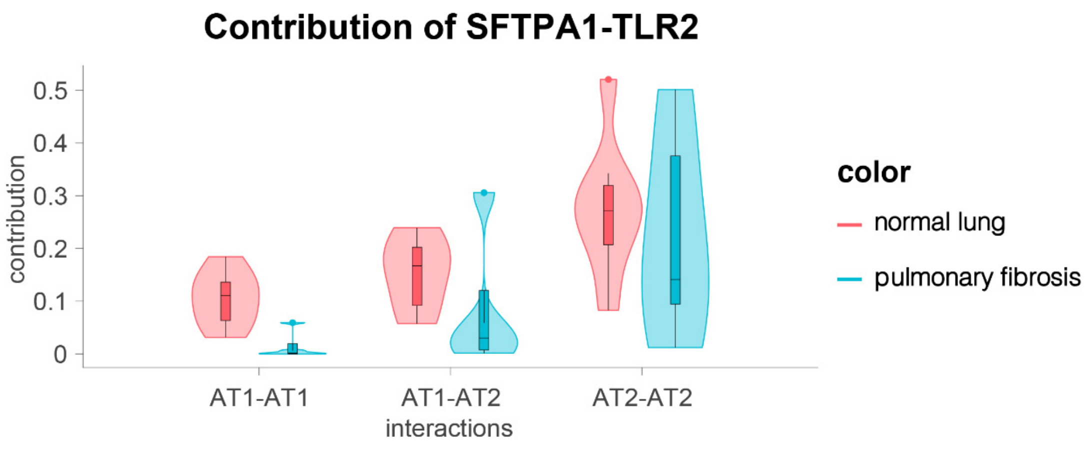

Fig. S4 Significant differences of the contribution of SFTPA1-TLR2 in normal alveoli compared with samples from patients with pulmonary fibrosis. *P*-values from left to right: 4.9×10^{-4}, 0.0056, and 0.0464 (rank-sum test, right side).
