# Supplementary material for: Reconstruction of cell spatial organization from single-cell RNA sequencing data based on ligand-receptor mediated self-assembly
Source: Cell Res. 2020 Jun 15;30(9):763–78. doi: 10.1038/s41422-020-0353-2 (PMC7608415; doi:10.1038/s41422-020-0353-2)
Supplement: Supplementary file 5 — Supplementary information, Fig. S5 [file 41422_2020_353_MOESM5_ESM.pdf]

Supplementary information, Figure S5

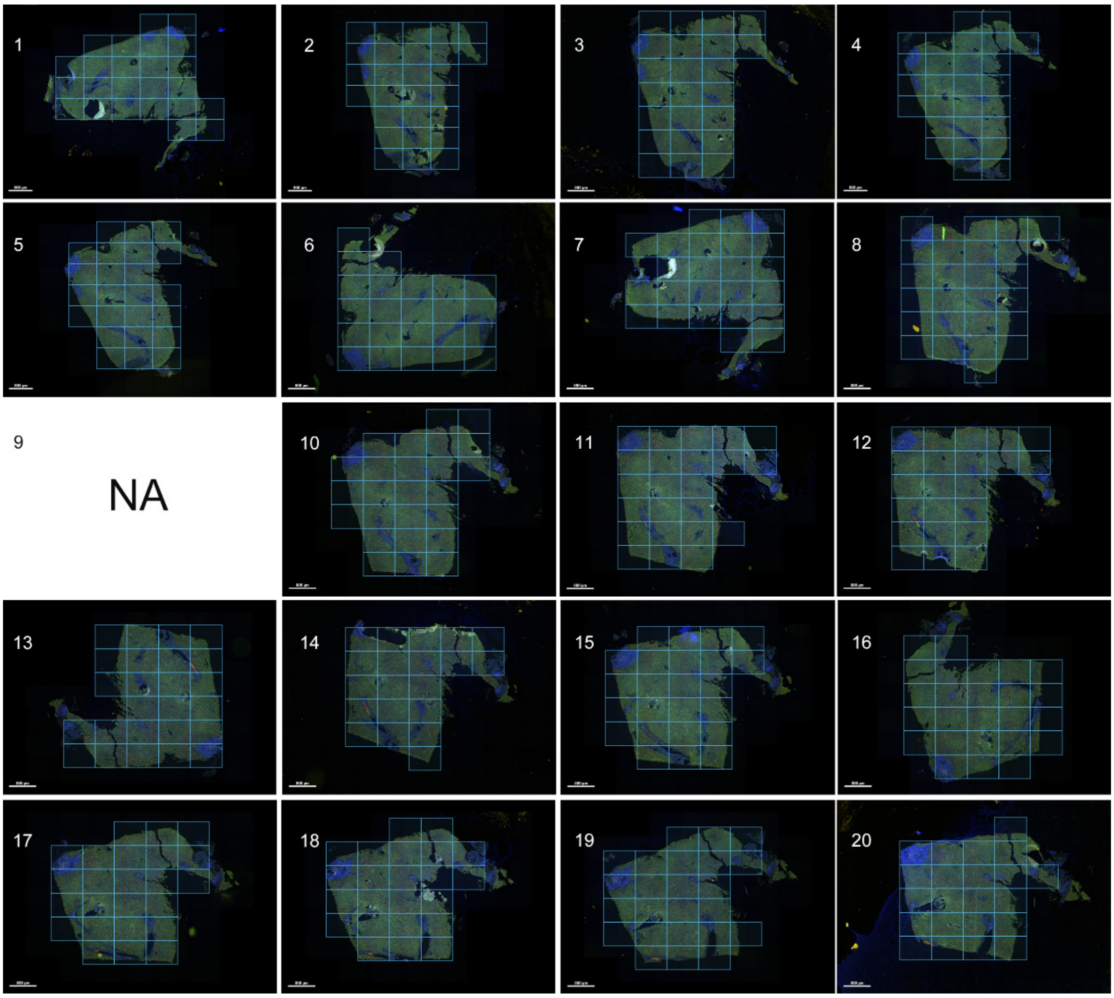

Fig. S5 IHC images of a centimeter-scale tumor sample from hepatocellular carcinoma. 20 consecutive 1 cm × 1 cm × 5 μm IHC images with the 9th staining failed. Treg: regulatory T cells ($Foxp3^+$); Tex: exhausted T cell ($PD\text{-}1^+$); CD8: $CD8^+PD\text{-}1^-$ T cells; cDC1: $CLEC9A^+$ dendritic cells; M: macrophages ($CD68^+$); O: other cells. The median distance of the 3rd nearest neighbor of all cells was used as the cutoff to determine whether two cells were spatially connected or not.
