# Supplementary material for: Reconstruction of cell spatial organization from single-cell RNA sequencing data based on ligand-receptor mediated self-assembly
Source: Cell Res. 2020 Jun 15;30(9):763–78. doi: 10.1038/s41422-020-0353-2 (PMC7608415; doi:10.1038/s41422-020-0353-2)
Supplement: Supplementary file 6 — Supplementary information, Fig. S6 [file 41422_2020_353_MOESM6_ESM.pdf]

Supplementary information, Figure S6

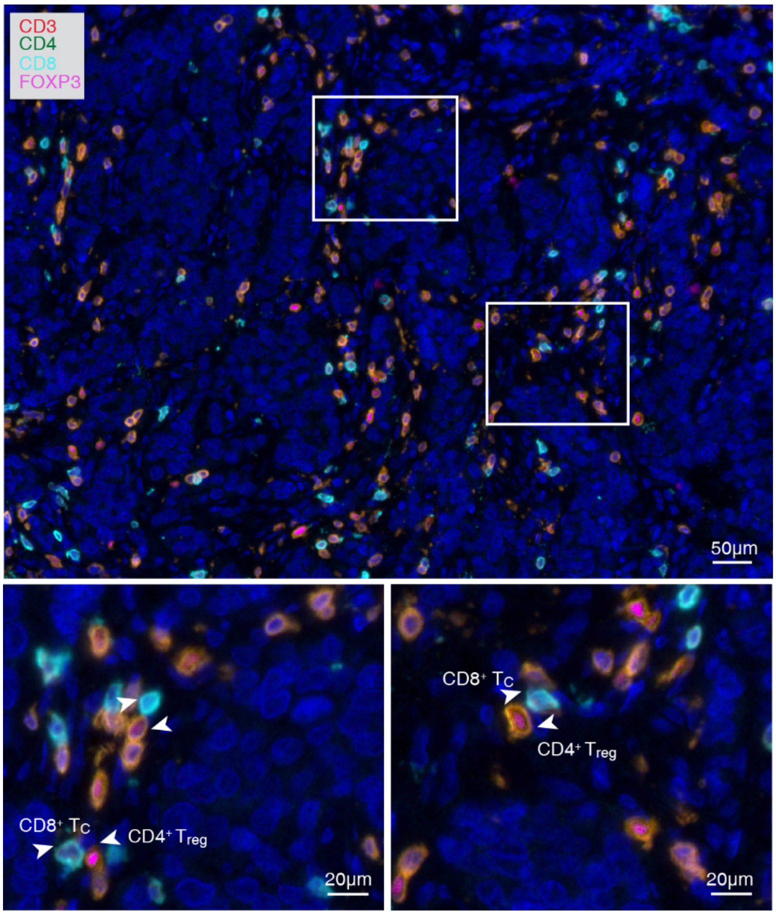

Fig. S6 Co-localization of Tregs (CD4$^+$FOXP3$^+$) and CD8$^+$ T cells revealed by IHC staining of a CRC tumor sample.
