# Supplementary material for: Reconstruction of cell spatial organization from single-cell RNA sequencing data based on ligand-receptor mediated self-assembly
Source: Cell Res. 2020 Jun 15;30(9):763–78. doi: 10.1038/s41422-020-0353-2 (PMC7608415; doi:10.1038/s41422-020-0353-2)
Supplement: Supplementary file 7 — Supplementary information, Fig. S7 [file 41422_2020_353_MOESM7_ESM.pdf]

Supplementary information, Figure S7

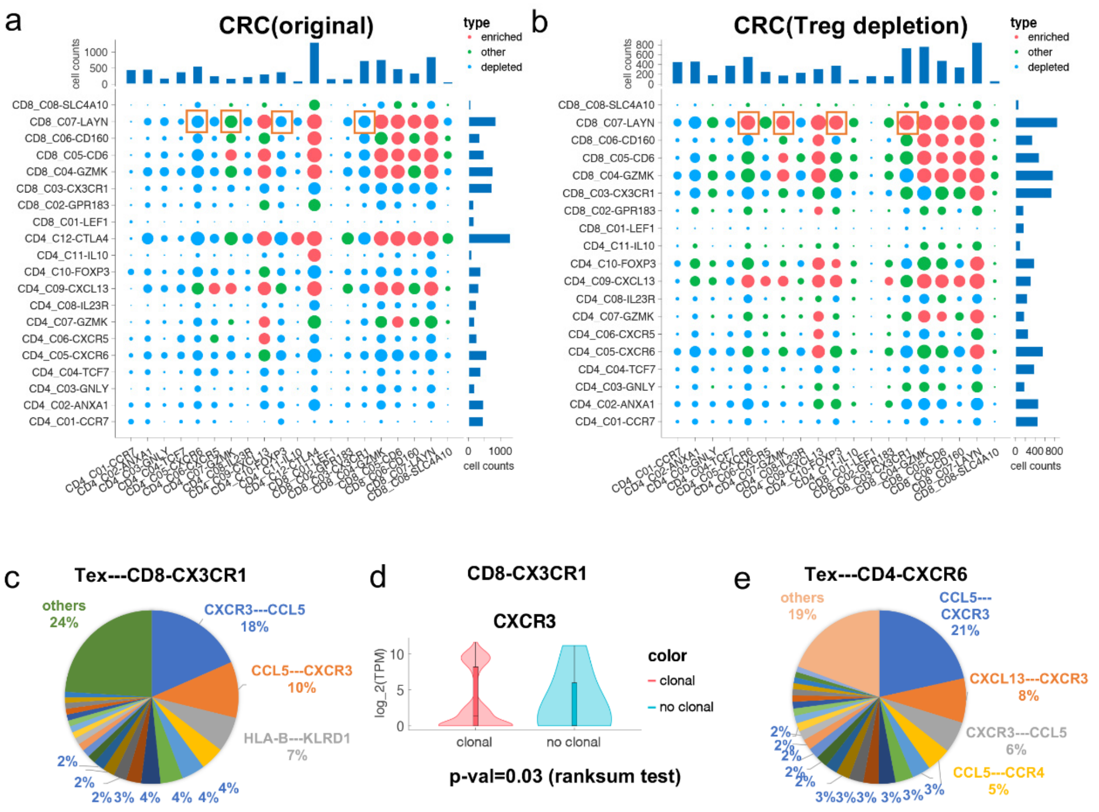

Fig. S7 Treg depletion enhanced the interactions of Texs with blood-enriched CD8-CX3CR1 and tissue-resident CD4-CXCR6 T cells revealed by CSOmap with *in silico* interference. a Statistical significance of cell-cell interactions of the original dataset of CRC. **b** Statistical significance of cell-cell interactions of the CRC dataset after Treg depletion. **c** The contributions of ligand-receptor pairs to Tex–CD8-CX3CR1 interactions. **d** CXCR3 was highly expressed in clonal CD8-CX3CR1 cells. **e** The contributions of ligand-receptor pairs to Tex–CD4-CXCR6 interactions.
