# Supplementary material for: Reconstruction of cell spatial organization from single-cell RNA sequencing data based on ligand-receptor mediated self-assembly
Source: Cell Res. 2020 Jun 15;30(9):763–78. doi: 10.1038/s41422-020-0353-2 (PMC7608415; doi:10.1038/s41422-020-0353-2)
Supplement: Supplementary file 8 — Supplementary information, Fig. S8 [file 41422_2020_353_MOESM8_ESM.pdf]

Supplementary information, Figure S8

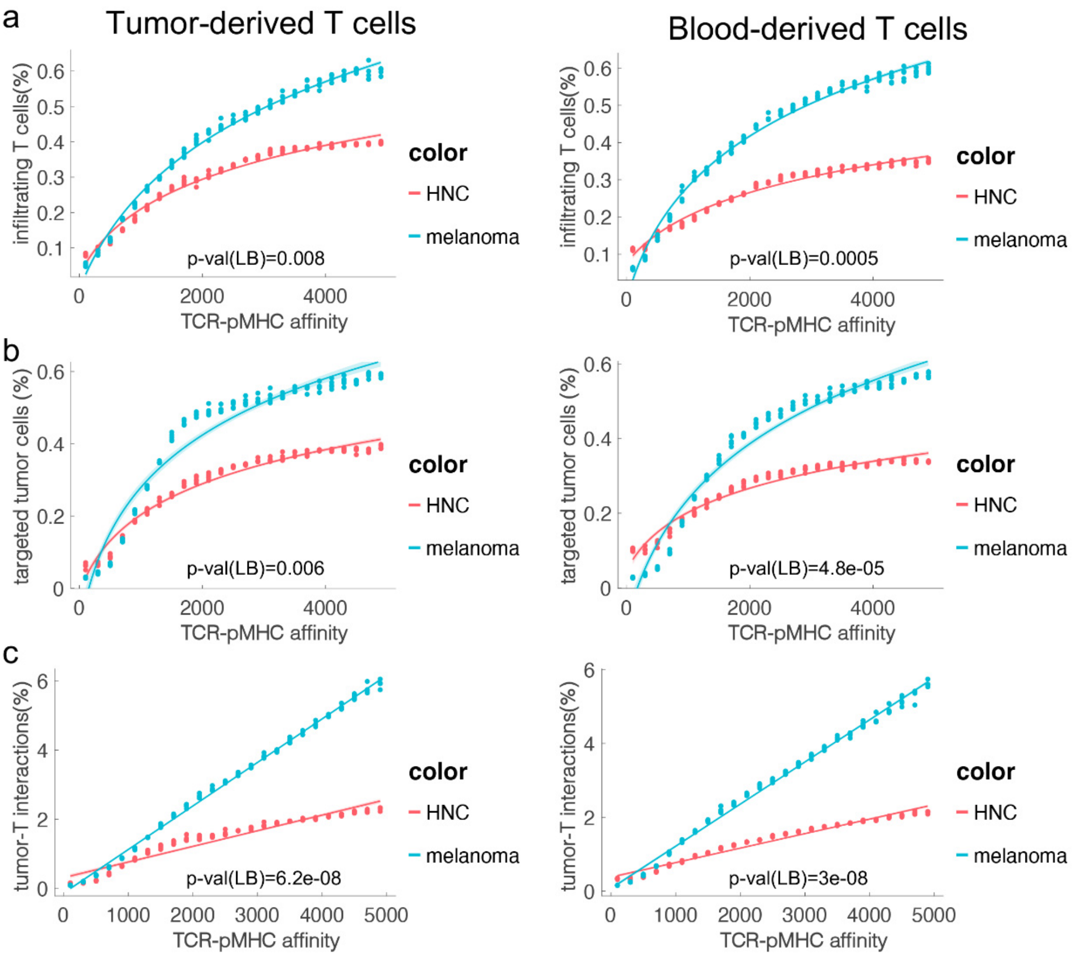

Fig. S8 Comparison of the effects of cancer characteristics on tumor-T interactions after adoptive T cell transfer. a The percentage of infiltrating T cells of the tumor-(left) and blood-derived (right) T cell transfer. **b** The percentage of targeted tumor cells of the tumor-(left) and blood-derived (right) T cell transfer. **c** The percentage of tumor-T interactions relative to the theoretical numbers of the tumor-(left) and blood-derived (right) T cell transfer. The statistical significance of TCR-pMHC affinity and the cancer characteristics to the percentages of infiltrating T cells, targeted tumor cells, and tumor-T interactions was evaluated by ANOVA analysis with repeated measures (by the ranova function of Matlab R2016b), and the lower bound (LB) of the *P*-values of the cancer types was displayed.
