# Supplementary material for: Reconstruction of cell spatial organization from single-cell RNA sequencing data based on ligand-receptor mediated self-assembly
Source: Cell Res. 2020 Jun 15;30(9):763–78. doi: 10.1038/s41422-020-0353-2 (PMC7608415; doi:10.1038/s41422-020-0353-2)
Supplement: Supplementary file 9 — Supplementary information, Fig. S9 [file 41422_2020_353_MOESM9_ESM.pdf]

Supplementary information, Figure S9

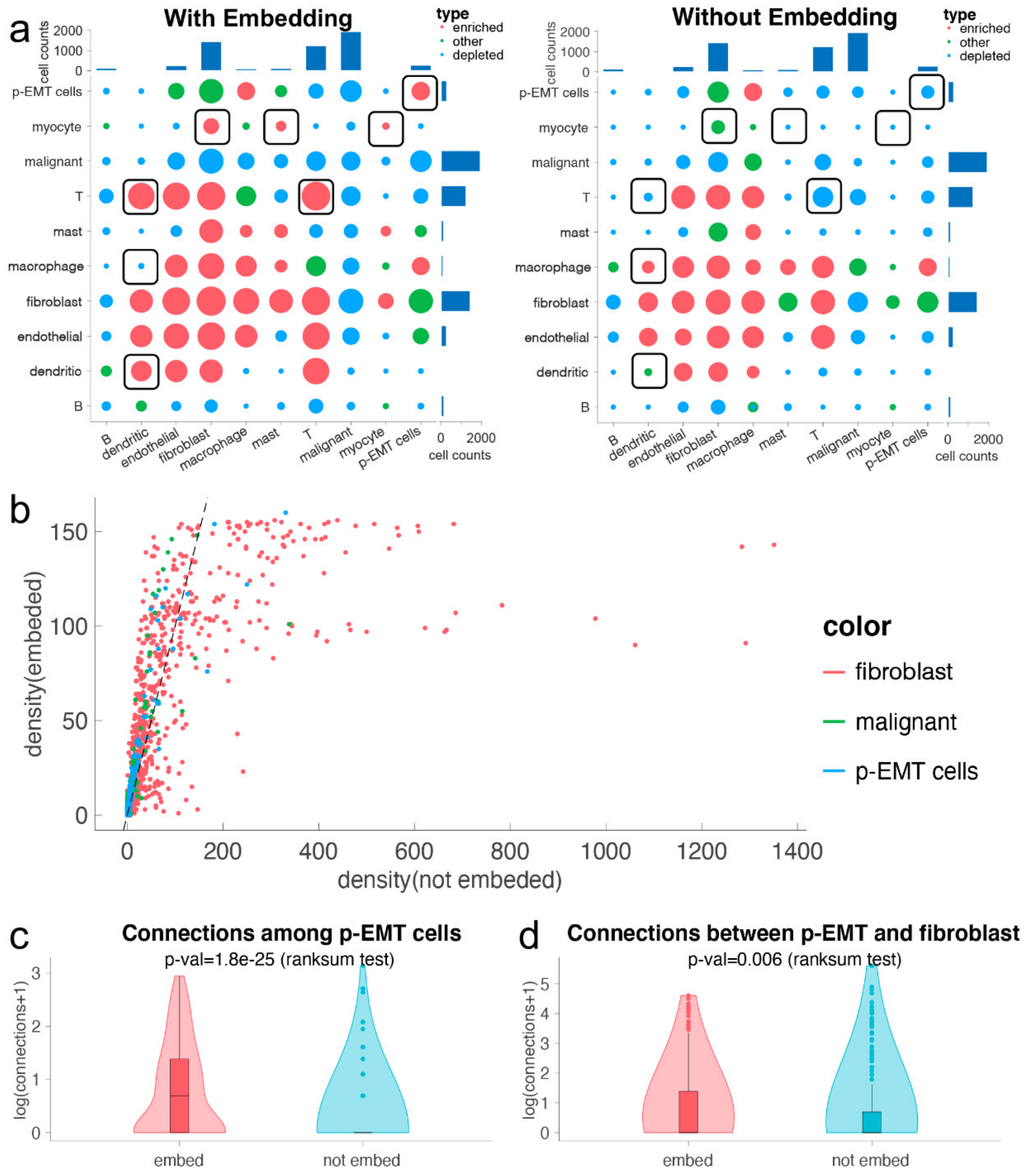

Fig. S9 Embedding cell-cell affinity matrix into three-dimensional space significantly improves spatial reconstruction accuracy (exemplified by the HNC dataset). a Statistical significance of the interactions among different cell types with/without embedding. Squared boxes indicate important differences, including the compact structure among p-EMT cells and interactions between T cells and dendritic

cells missed by the without-embedding method. **b** Extremely high density for fibroblast cells in the without-embedding reconstruction due to the lack of consideration of the limitations of realistic space. **c, d** Tight connections among p-EMT cells and between p-EMT cells and fibroblasts were revealed by the with-embedding method rather than without embedding.
