# Supplementary material for: Reconstruction of cell spatial organization from single-cell RNA sequencing data based on ligand-receptor mediated self-assembly
Source: Cell Res. 2020 Jun 15;30(9):763–78. doi: 10.1038/s41422-020-0353-2 (PMC7608415; doi:10.1038/s41422-020-0353-2)
Supplement: Supplementary file 10 — Supplementary information, Fig. S10 [file 41422_2020_353_MOESM10_ESM.pdf]

Supplementary information, Figure S10

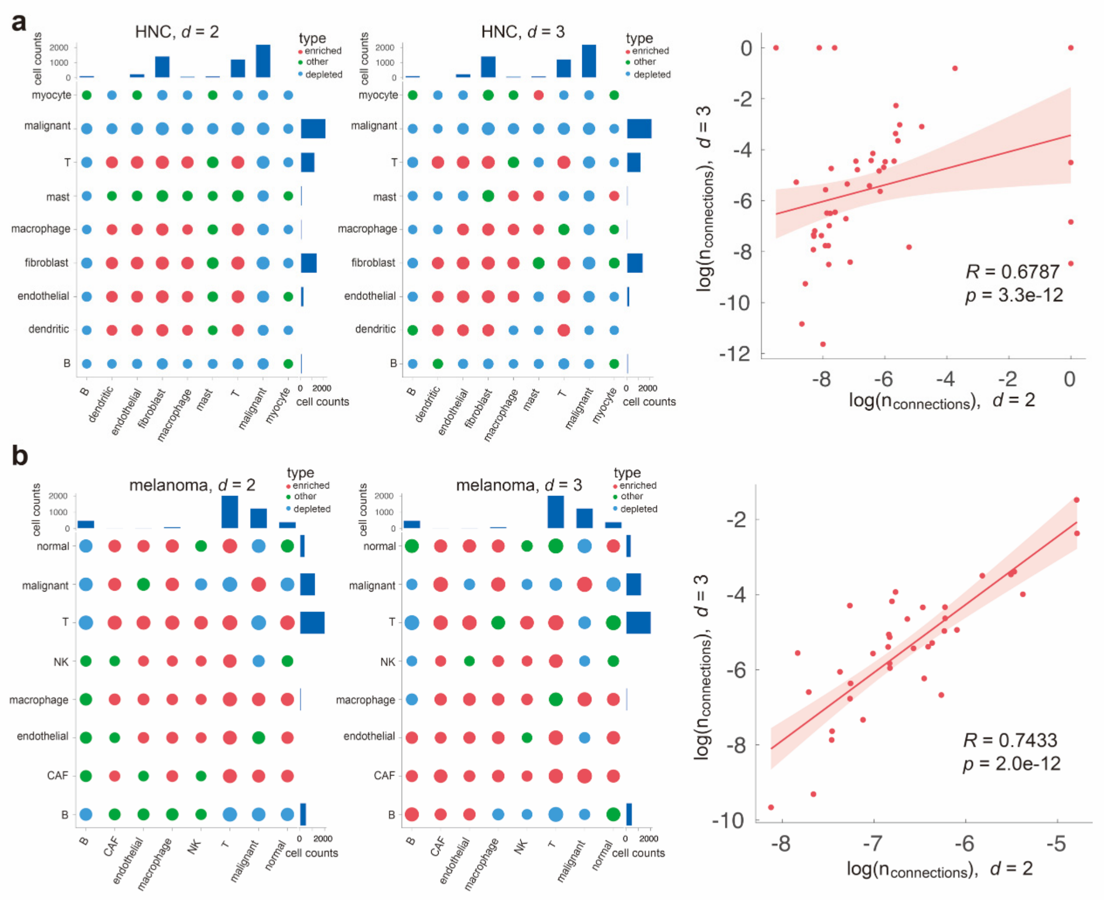

Fig. S10 The parameter of dimension number has critical impacts on the predictions of CSOmap. As real biological tissues and organs are inherently of 3D, the default dimension parameter of CSOmap is set to 3. Spearman correlation was applied to evaluate the consistence between different parameters. Each dot in the right scatter plots represents one dot in the left matrix, i.e., the observed connections between the row clusters and column clusters normalized by the cluster sizes.
