# Supplementary material for: Reconstruction of cell spatial organization from single-cell RNA sequencing data based on ligand-receptor mediated self-assembly
Source: Cell Res. 2020 Jun 15;30(9):763–78. doi: 10.1038/s41422-020-0353-2 (PMC7608415; doi:10.1038/s41422-020-0353-2)
Supplement: Supplementary file 11 — Supplementary information, Fig. S11 [file 41422_2020_353_MOESM11_ESM.pdf]

Supplementary information, Figure S11

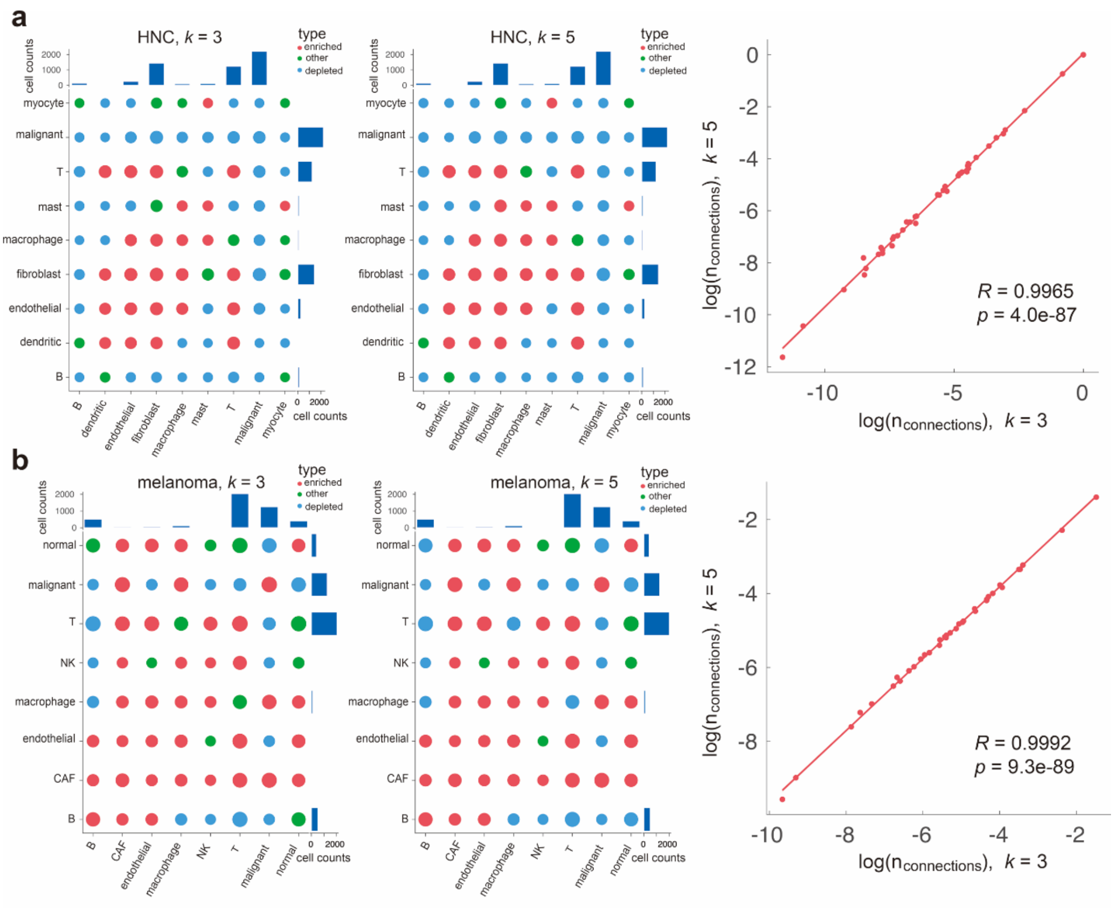

Fig. S11 Predictions of CSOmap are robust to the selection of cutoffs (*k*) determining whether two cells are connected in the pseudo-space. The median distance of the *k*-th nearest neighbor of all cells was used to determine whether two cells were connected or not. Spearman correlation was applied to evaluate the consistence between different parameters. Each dot in the right scatter plots represents one dot in the left matrix, i.e., the observed connections between the row clusters and column clusters normalized by the cluster sizes.
