# Supplementary figures and images for: Reconstruction of cell spatial organization from single-cell RNA sequencing data based on ligand-receptor mediated self-assembly
Source: Cell Res. 2020 Jun 15;30(9):763–78. doi: 10.1038/s41422-020-0353-2 (PMC7608415; doi:10.1038/s41422-020-0353-2)

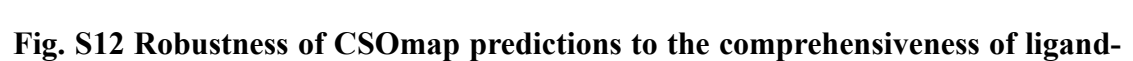

Supplement: Supplementary file 12 — Supplementary information, Fig. S12 [file 41422_2020_353_MOESM12_ESM.pdf]
